# *Mycobacterium bovis* infection at the interface between domestic and wild animals in Zambia

**DOI:** 10.1186/1746-6148-8-221

**Published:** 2012-11-14

**Authors:** Mudenda B Hang’ombe, Musso Munyeme, Chie Nakajima, Yukari Fukushima, Haruka Suzuki, Wigganson Matandiko, Akihiro Ishii, Aaron S Mweene, Yasuhiko Suzuki

**Affiliations:** 1 School of Veterinary Medicine, University of Zambia, P. O. Box 32379, Lusaka, Zambia; 2 Hokkaido University Research Center for Zoonosis Control, Kita-20, Nishi-10, Kita-ku, 001-0020, Sapporo, Japan; 3 Zambia Wildlife Authority, Private Bag 001, Chilanga, Zambia; 4 JST/JICA-SATREPS, Tokyo, 120-8666, Japan

**Keywords:** Bovine tuberculosis, Cattle, *Mycobacterium bovis*, Strains, Wildlife, *Kobus leche Kafuensis*

## Abstract

**Background:**

In Zambia, the presence of bovine tuberculosis in both wild and domestic animals has long been acknowledged and mutual transmission between them has been predicted without any direct evidence. Elucidation of the circulating *Mycobacterium bovis* strains at wild and domestic animals interphase area in Zambia, where bovine tuberculosis was diagnosed in wildlife seemed to be important.

**Results:**

A PCR identified 15 and 37 *M. bovis* isolates from lechwe and cattle, respectively. Spoligotype analysis revealed that *M. bovis* strains from lechwe and cattle in Kafue basin clustered into a major node SB0120, where isolates outside the Kafue basin clustered into different nodes of SB0131 and SB0948. The comparatively higher variety of strains in cattle compared to lechwe elucidated by Mycobacterial Interspersed Repetitive Units–Variable Number Tandem Repeats analyses are consistent with cattle being the probable source of *M. bovis* in wild and domestic animals interphase area in Zambia.

**Conclusions:**

These results provide strong evidence of *M. bovis* strains transfer between cattle and lechwe, with the latter having developed into a sylvatic reservoir host.

## Background

Majority of the mycobacterial species that cause human and animal tuberculosis are grouped together as members of the *Mycobacterium tuberculosis* complex (MTC) [1,2]. This *Mycobacterium tuberculosis* complex includes very closely related species of mycobacteria among them: *M. tuberculosis, M. africanum, M. microti, M. bovis, M. caprae, M. canettii* and *M. pinnipedii*[1]. Although *M. tuberculosis* infection is the most common cause of human tuberculosis, part of other proportion of cases are due to *M. bovis*[3,4].

Zoonotic tuberculosis is caused mainly by *M. bovis* that has been shown to have a very wide host range [4-6]. The specie has been documented throughout the world with a similar impact in terms of disease occurrence [5]. In Zambia, BTB is not homogenously distributed, however, high prevalence rates have been recorded within and around the Kafue basin where there is extensive overlap in terms of grazing land for both wild and domestic animals [7-9]. Additionally, the Kafue lechwe antelopes (*Kobus leche Kafuensis*) found in the Kafue basin have been described as feral reservoirs of BTB in Zambia [10,11]. The disease has a historical presence in the Kafue basin that predates the identification of the area as a protected ecosystem and Ramsar Site no.530 [12]. Despite the continued reduction in annual rainfall figures under the effects of global warming, the Kafue basin still remains as one of the few lucurstrine wetland ecosystems in Zambia and Africa, supporting a surging cattle population estimated at 300,000 animals, [13] at a carrying density of 50 animals per square kilometre and approximately 38,000 lechwe antelopes [12] on a 6,000 square kilometre wetland [14]. The area is characterised by high BTB with a herd level prevalence of around 50%, whereas a comparatively lower herd prevalence averaging 5.6% has been determined in areas outside the basin [7,15]. Likewise, the corresponding Kafue lechwe antelopes have been shown to have a higher BTB prevalence rate [11,16], raising questions on a possible interspecies transmission of the disease between cattle and Kafue lechwe antelopes. This is however hampered by the lack of direct evidence to conclusively ascertain this assertion.

Sequencing of the whole genome of the members of MTC [17-21] has shown a high level of sequence homogeneity among the members (99.95%). Thus a careful and detailed comparative exploration into the individual genomes of the members of this complex was employed to mine out significant differences for diagnostic capabilities. Comparative genome analysis informed us that *M. bovis* has a smaller genome compared with *M. tuberculosis*[1]. Furthermore, *M. bovis* has over time lost off some genes compared to *M. tuberculosis.* These genomic insertion-deletions are commonly referred to as Regions of difference (RD) and have been used in speciation of members of this complex as well as in explaining the evolution of the MTCs [1,17,19]. Spoligotyping diagnostic technique highlights intra species differences determined by the loss of spacers at a direct repeat region in MTCs, thereby creating a fingerprint typical of a particular specie [22]. Additionally, it is a more rapid and specific method of MTC speciation apart from being less laborious compared with biochemical, phenotypic and IS*6110*-restriction fragment length polymorphism (RFLP) analysis [22]. Spoligotyping results are very practical and reproducible across different laboratories internationally [22,23]. The technique has developed considerably and it is marked by a system of nomenclature and strain data capture and identification with a huge geographical and epidemiological relevance worldwide [24]. Most strains of *M. bovis* have one copy of IS*6110* and spoligotyping is in general more discriminative when used with methods based on PCR amplification of the loci containing variable number tandem repeats (VNTRs) [25,26].

The target of this study was to molecularly characterize a population sample of *M. bovis* from cattle and kafue lechwe antelopes in Zambia to determine the genetic diversity and relatedness of the isolates from domestic animals and wildlife.

## Results and discussion

### Isolation and confirmation of *M. bovis* by MTCD-MPCR

A total of 315 specimens from cattle and 75 from lechwe antelopes were analysed to initially determine the prevalence of MTC species. The samples were collected based on observations of gross pathological lesions upon examination. The observations included generalized lesions involving the lungs, pleural and mediastinal lymph nodes in both lechwe and cattle. These tuberculous lesions were observed by other workers who were investigating gross pathological distribution of tuberculous lesions in both cattle and lechwe [9,27,28]. From the samples analysed, 52 MTC strains (Table 1) were obtained with 37 isolates from 315 cattle and 15 from 75 lechwe. Following isolation *M. bovis* was confirmed by screening using the MTCD-MPCR analysis as previously observed [11,29]. The MTCD-MPCR technique is very useful in the differentiation of MTC at whatever level of diagnosis as it is simple and specific [29-31]. The presence of *M. bovis* in wildlife may translate into a perpetual focus of the disease [10] considering that BTB control in wild animals is a very difficult undertaking [32,33]. The only workable solution would be to intensify BTB testing in domestic animals so that reactors are removed. Furthermore traditional cattle herders must also be informed of the dangers of grazing their animals in areas where lechwe antelopes are present.

**Table 1:** **Results of the MTCD-MPCR of the isolated *Mycobacterial *isolates from cattle and lechwe**

**Animal species**	**Total sampled**	**MTCD-MPCR *M. bovis +ve***
Cattle	315	37
Lechwe antelope	75	15
Total	390	52

### Spoligotyping and Multiple locus variable number of tandem repeats analysis

Spoligotyping of the 52 *M. bovis* isolates revealed their molecular clonality (Table 2). Two major spoligotype patterns (SB0120 and SB0131) were observed accounting for 36 isolates (69.2%), and 15 isolates (28.8%) cattle and lechwe respectively. Two isolates not identifiable with the two major clusters was given an SB0948 under the global spoligotype patterns diversity provided by the international data base on spoligotyping [24]. This only accounted for 4% of the observed isolates. *M. bovis* strains from both cattle and lechwe of the Kafue basin were found to share the same spoligotype (SB0120). This spoligotype was previously reported to be the major strain circulating in cattle around the Kafue basin, although by that time, no strains were determined from wild animals [34]. *M. bovis* strains isolated outside the Kafue basin were found to share a different cluster (SB0131). All the 52 isolates lacked spacers 3, 9, 16 and 39 to 43, a characteristic feature that distinguishes *M. bovis* from *M. tuberculosis*[22]. Molecular studies have previously shown that clonality implies active transmission of disease [35]. Thus our results in the Kafue basin where there was high clonality between isolates from lechwe and cattle, suggest an active transmission of *M. bovis* between the two animal species. The spoligotyping results showed that SB0131, clustered outside the Kafue basin suggesting that none of the lechwe antelopes harboured *M. bovis* with such a spoligo pattern.

**Table 2:** Spoligotyping results by area of origin across animal species

**Animal species**	**Area of origin**	**Strain type**	**Clade**	**STI**	**No of isolates**
Lechwe	Blue lagoon National park	SB0120	BOVIS 1_BCG	482	4
	Lochinvar National park	SB0120	BOVIS 1_BCG	482	11
Cattle	Namwala (Southern Zambia)	SB0120	BOVIS 1_BCG	482	20
		SB0131	BOVIS 1	594	4
	Lusaka (Central Zambia)	SB0131	BOVIS 1	594	1
	Chongwe (Central Zambia)	SB0120	BOVIS 1_BCG	482	1
		SB0131	BOVIS 1	594	1
	Kabwe (Central Zambia)	SB0131	BOVIS 1	594	8
	Mumbwa (Central Zambia)	SB0948	No data	New	1
	Nampundwe (Central Zambia)	SB0948	No data	New	1
Total					52

**Figure 1:**
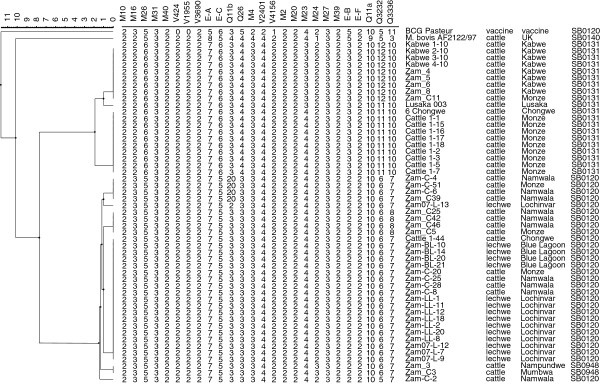
**Dendrogram based on the *M. bovis *MLVA clusters distribution data.** The major spoligotype SB0120 observed in cattle and lechwe are presented with varying MLVA types. The spoligotype SB0131 is also indicated with 3 MLVA types.

MLVA analysis identified 2 distinct clusters, which are corresponding to either SB0131 or SB0120 (Figure 1). Within the cluster possessing the spoligo pattern SB0131, there were 2 groups and 1 strain distinguishable one another by 2 loci, MIRU23 and QUB3232. The cluster possessing the Spoligotype SB0120 was differentiated in 6 groups. Nineteen strains, 14 from lechwe and 5 from cattle, consisted of a major group and other 5 groups or singletons were discriminated from the major group by a single MLVA locus difference each. The spoligotype SB0948 seems to be a descendant pattern of SB0120 lacking spacer 1 and also clustered in SB0120 group with 1 locus (MIRU24) difference. The high isolation frequency of spoligotype SB0120 in cattle and lechwe conforms to the finding of previous works from Zambia [34] and may demonstrate dominance of this spoligotype despite the small sample size. The SB0120 spoligotype has a considerable degree of geographical dispersion in Zambia and other African countries such as Algeria [36] and South Africa [37]. This spoligotype is also common in continental Europe [37-39]. Such a strain could have been introduced through livestock development schemes of trying to improve African beef local breeds. This finding has been highlighted by different workers [36,40,41]. Spoligotype SB0131 has been reported in this work for the first time in Zambia. This spoligotype has been documented in South Africa [42], which has a close livestock trade link with Zambia.

**Figure 2:**
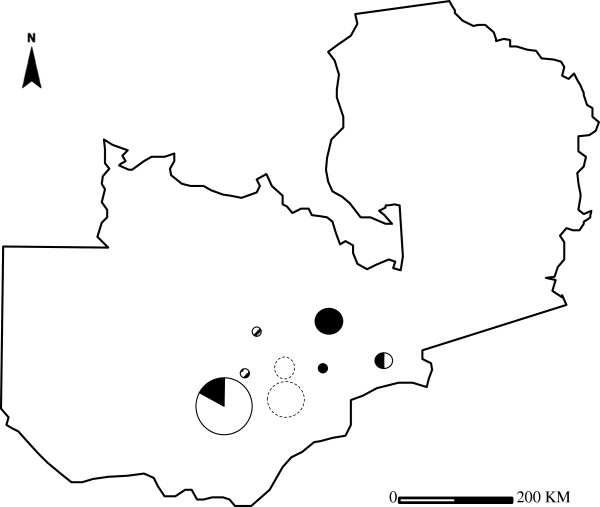
**Spread of *M. bovis *strains in Zambia.** Circle and dotted circle indicates the *M. bovis* from cattle and lechwe, respectively. White portion in circles indicates the *M. bovis* isolates with spoligotype SB0120, Black portion of circles indicates the *M. bovis* isolates with spoligotype SB0131, and the shaded circle indicates the *M. bovis* with spoligotype SB0948. Size of circle indicates the strain number.

To our knowledge, this is the first study conducting molecular characterization of *M. bovis* strains from cattle and lechwe in Zambia. However, routine studies have already previously revealed the presence of BTB in these animals [7,11,16,34]. In this study, results have shown genetic relatedness between *M. bovis* in cattle and lechwe antelopes of the Kafue basin. It is important to note that the area around Lochnivar National park was used for ranching purposes by the early settlers from South Africa. This area was only gazetted as an animal sanctuary in 1972. It is the only place where Kafue lechwe antelopes antelopes are confined. The animals are semi-aquatic living in large groups near water and as such are confined to the area shown in Figure 2 (dotted circle).

Characterization of *M. bovis* strains based on molecular tools is important in understanding the epidemiology of BTB [25,36,41]. These results are significant in understanding the transmission and dispersion of *M. bovis* strains within Zambia (Figure 2), given the high level of internal migration by the local people from the Southern part of the country to the Central and Northern regions of Zambia with their cattle. This type of internal migration may lead to the dispersion of the SB0120 strain which in this present study was found confined to the Southern regions of Zambia.

## Conclusion

The current study has described the possible source of *M. bovis* in wildlife and the transmission of limited strains of *M. bovis* between cattle and lechwe. The identified maintenance and spread of *M. bovis* may become a dynamic and highly active process considering that human activities allow movement of cattle from one location to another.

## Methods

### Study area and sampling

**Figure 3:**
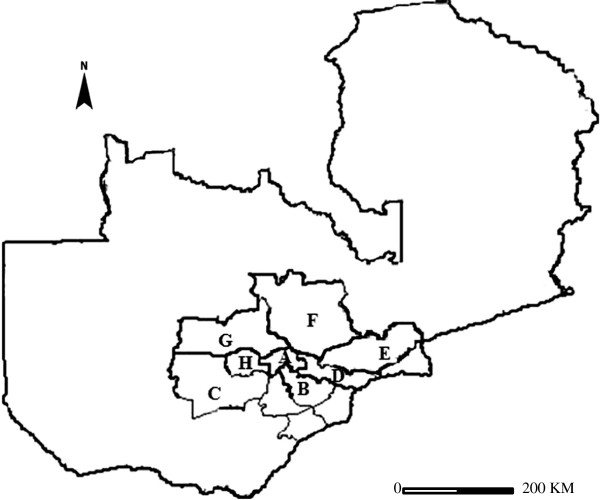
**Study area in Zambia.A**: Blue lagoon National park, **B**: Lochnivar National park, **C**: Namwala, **D**: Lusaka, **E**: Chongwe, **F**: Kabwe, **G**: Mumbwa, **H**: Nampundwe. **A**, **B**, **C**, **G** and **H** are an interphase area consisting of a wildlife sanctuary and a traditional cattle grazing area; while Area **D**, **E** and **F** are a farming area consisting of commercial and subsistence cattle farms.

All the samples used in this study were collected in Zambia from 2006 to 2010. The bulk of the samples were obtained at abattoirs from cattle of the Kafue Basin and from other areas supplying cattle to the main abattoirs of Lusaka, the capital city of Zambia (Figure 3). The animals were coming from the Southern province and Central areas of Zambia. Carcasses were followed along the slaughter line and screened for any visible tuberculous lesions. All animals suspected to have tuberculous lesions were examined further by collecting samples for laboratory analysis. The wildlife samples were obtained from Kafue lechwe antelopes, which were sampled over the same period as cattle through a special research license from the Zambia Wildlife Authority. The sample size depended on the number of animals allocated for scientific studies with the selection being done at random by gun stunning. The animals were collected from Blue lagoon national park (Figure 3: area A) and Lochnivar national park (Figure 3: area B). The animals were examined for gross lesions according to the standard post mortem examination procedures [43]. The organs and tissues with suspected TB lesions were collected together with accompanying demographical data of area of origin, type of organ or tissue as well as the type of gross pathological postmortem disposition. These specimens were placed in sterile self-zipping histopathological bags, placed into a cooler box with ice packs before transportation to the laboratory for analysis.

#### Cultivation of Mycobacterial and DNA extraction

The collected samples of TB suspect were analyzed and cultured for growth as previously described [16]. Briefly, the tissues were trimmed of fat and then a 500 mg sample was minced with sterile scissors and homogenized in a sterilized glass homogenizer. A milliliter of phosphate buffer (pH 6.8) was added and thoroughly mixed after which 1 mL of 5% sodium hydroxide was added. After incubation for 15 min at room temperature, 10 mL of phosphate buffer was added and then centrifuged at 1500 *xg* for 20 min. The pellet was collected and then resuspended in a final volume of 0.5 mL of phosphate buffer which was then used for inoculation onto 2% Ogawa medium. Bacterial growth was then monitored up to 8 weeks at 37°C. The resulting cultures were tentatively identified as probable *Mycobacterium tuberculosis-*complex by their slow growth and colony morphology. Purity and acid-fastness of the colonies were checked by Ziehl Neelsen staining. DNA was extracted from Mycobacterial colonies using DNAzol reagent (Invitrogen, Carlsbad, CA, USA) and mechanical disruption as previously described [44] according to the manufacturer’s instructions and dissolved in 50 μL TE buffer consisting of 10 mM Tris/HCL (pH 8.0) and 1 mM EDTA.

#### MTC discrimination by multiplex PCR

The *Mycobacterium tuberculosis* complex-discriminating multiplex PCR (MTCD-MPCR) targeting genetic regions *cfp32* (a specific gene for MTC), RD9 (region of difference 9; seen only in *M. tuberculosis* and *M. canettii*) and RD12 (region of difference 12; deleted in *M. bovis*, *M. caprae* and *M. canettii*) was used for species differentiation according to the previously publication [27]. The reaction mixture consisted of 7.4 μL H_2_0, 2 μL 10 x Taq buffer, 2 μL dNTPs (2.5 mM each), 0.2 μL Taq (Takara Bio Inc, Shiga, Japan), 1 μL of DNA sample, 2.2 μL of 10 μM cfp32 primers, 0.7 μL of 5 μM RD9 primers and 0.8 μL of 5 μM RD 12 primers. The PCR was performed using the following program: denaturation for 1 min at 98°C followed by 35 cycles of 5 sec at 98°C, 20 sec at 58°C and 1 min at 68°C with final elongation at 72°C for 5 min in a thermal cycler (iCycler, Bio-Rad Laboratories Inc., Hercules, CA). The PCR products were separated by electrophoresis in a 2% agarose gel in TAE buffer, and then visualized after staining with ethidium bromide.

#### Spoligotyping of *M. bovis* isolates using micro-spoligoarrays

Spoligotyping was performed according to the procedure by Kamerbeek and co-workers with slight modifications [22]. Forty-three spacer-sequence probes were covalently bound to the membrane (Pall Co., NY, USA). The primers used were DRa (GGTTTTGGGTCTGACGAC) and DRb (CCGAGAGGGGACGGAAAC). A hot start PCR was done by mixing 1 μL each of the 10 μM primer, 7.3 μL H_2_O, 3 μL of 5 x colorless Go Taq buffer (Promega™, Fitchburg, WI), 1.5 μL of PCR DIG Labeling Mix (Roche), 0.2 μL of Go Taq DNA Polymerase (5 units/μL; Promega) and 1 μL of DNA sample in 15 μL total reaction mix per tube. The PCR reaction was initiated by denaturation at a temperature of 98°C for 1 min, followed by 40 cycles of 98°C for 5 sec, 55°C for 10 sec and 72°C for 30 sec with a final elongation step at 72°C for 1 min in a thermal cycler. The 500 times diluted PCR product in hybridization buffer was heat denatured at 95°C for 5 minutes and immediately cooled on ice to leave the DNA single stranded. Hybridization was performed by placing the nitrocellulose membranes (Pall Co., NY, USA) for incubation at 60°C for 1 hour. After which, the membrane was washed in TBST-E (0.1% Tween-20 and 1mM EDTA-2Na in TBS) at 60°C for 1 min and then10 min, finally 1 min. The membrane was then dried at room temperature. DIG on the nitrocellulose membranes were reacted with a 1000 times diluted Ant-Digoxigenin-POD [poly], Fab fragments (Roche), with TBSTE-E at room temperature for 30 min. Then the membranes were sequentially washed in TBST-E at room temperature for 1 min, 10 min and 1 min. Then, POD on the membranes was detected by TMB solution (TMB Peroxidase Substrate Kit, Vector Labs Inc™, Burlingame, CA) according to the manufacturer’s protocol.

#### Multiple locus variable number of tandem repeats (MLVA) assay of *M. bovis* isolates

The isolates were further genotyped by PCR amplification using primers targeting 26 VNTR loci [45]. Two different PCR reaction mixtures were conducted according to the loci. Locus MIRU10, 16, 24, 26, 27, 39, 40, ETR-B, F, VNTR-424, 3690 were done under betaine 1.0 M, whilst locus MIRU2, 4, 20, 23, 31, ETR-A, C, QUB-11a, 11b, 26, 3232, 3336, VNTR-1955, 2401, 4156 were performed under GCII buffer (Takara). The PCR reaction mixture under 1.0 M betaine buffer was conducted in a mixture consisted of 6.3 μL H_2_O, 0.4 μL of dNTP (10 mM each), 3.0 μL of 5x colorless Go Taq buffer, 3.0 μL of betaine (5 M), 0.6 μL of Primer 1 and 0.6 μL of Primer 2, 0.1 μL of Go Taq (5 units/ μL) DNA Polymerase, and 1 μL of DNA sample. An initial denaturation step of 95°C for 5 min was followed by 32 cycles at 95°C for 15 sec, 58°C for 20 sec and 72°C for 1 min with a final elongation step at 72°C for 1 min in a thermalcycler. The PCR reaction mixture under the GCII buffer consisted of 4.8 μL H_2_O, 0.4 μL of dNTP (10 mM each), 7.5 μL of 2 x GCII buffer, 0.6 μL of primers, 0.1 μL of Go Taq (5 units/ μL) DNA Polymerase (Promega), and 1 μL of DNA sample. An initial denaturation step at 95°C for 5 min was followed by 32 cycles of 95°C for 15 sec, 50°C for 20 sec and 72°C for 45 sec with a final elongation step at 72°C for 1 min in a thermalcycler. All the samples were electrophoresed on a 2% agarose gel to identify the repeat numbers.

## Abbreviations

PCR: Polymerase chain reaction; BTB: Bovine tuberculosis; MTCD-MPCR: *Mycobacterium tuberculosis* complex-discriminating multiplex PCR; MIRU-VNTR: Mycobacterial interspersed repetitive units–variable-number tandem repeats; RD: Regions of difference.

## Competing interests

All authors have no competing interests.

## Authors’ contributions

HBM and MM undertook sample collection, laboratory experiments, analyzing the results and drafting the manuscript. CN contributed the data analysis. YF, HS and AI were responsible for laboratory experiments. WM contributed to the design of field data collection. ASM and YS contributed to the design, writing of the manuscript and coordinated the study. All authors read and approved the manuscript.

## Authors’ information

HBM, MM, CN, WM and ASM are veterinarians.
